# New program for identification of child maltreatment in emergency department: preliminary data

**DOI:** 10.1186/s13052-016-0275-2

**Published:** 2016-07-13

**Authors:** Gregorio P. Milani, Federica A. Vianello, Barbara Cantoni, Carlo Agostoni, Emilio F. Fossali

**Affiliations:** Pediatric Emergency Department, Fondazione IRCCS Ca’ Granda, Ospedale Maggiore Policlinico, Milan, Italy; Pediatric Emergency Department and SITRA Pediatric Area, Fondazione IRCCS Ca’ Granda, Ospedale Maggiore Policlinico, Milan, Italy; Department of Pediatrics, IRCCS Foundation Ca’ Granda, Ospedale Maggiore Policlinico, University of Milan, Milan, Italy; Fondazione IRCCS, Ca’ Granda, Ospedale Maggiore Policlinico, Via della Commenda 9, 20122 Milan, Italy

**Keywords:** Child maltreatment, Emergencies, Interdisciplinary health team

## Abstract

Early detection of child maltreatment in pediatric emergency department is one of the most important challenges for the Italian and European medical care system. Several interventions have been proposed, but results are often unquantifiable or inadequate to face this problem. We promoted an educational program and built up an interdisciplinary team to improve the identification and management of maltreated children. Aim of this study is to report preliminary results of these interventions. Meetings structured with lecture-based teaching and case-based lessons were focused on identification and management of maltreatment cases. An interdisciplinary team with forensic physicians, dermatologists, orthopedics, radiologists, gynecologists, oculists, psychologists and psychiatrics, was created to manage children with suspected diagnosis of maltreatment. We analysed the characteristics of subjects diagnosed after these interventions and their number was compared with the one in the two previous years. An increased rate of diagnoses of 16.9 % was found. Results of the reported program are encouraging, but many efforts are still mandatory to improve the child maltreatment identification in emergency departments.

Child maltreatment is an overwhelming worldwide problem that can cause serious injuries up to death, and long-term consequences for victims, their family and society in general [1]. Many efforts have been made to increase the detection rate of child maltreatment in emergency departments, but the results are often unquantifiable or inadequate [2]. We promoted an educational program, focused on the identification of maltreatment cases in pediatric emergency department, and a dedicated interdisciplinary team to improve the management of such cases was built up. The educational program began in October 2011 and lasted for 10 weeks (one meeting of 4–6 hours/week). Nursing and medical staff working in the pediatric emergency department Fondazione Ca’ Granda Ospedale Maggiore Policlinico, Milan and other specialists, potentially involved in the identification of maltreatment cases, were asked to participate. The meetings (lecture-based teaching and case-based lessons) aimed to promote the abilities to identify cases of maltreatment and their management. The first four meetings were focused on history taking and identification of risk factors (e.g. psychiatric disorders in a member of the family). Other 4 weeks were addressed to identify types of injuries consistent with maltreatment. Finally, two meetings were addressed to organize the intervention of the team in suspected cases and to discuss their management. Besides the medical and nurse staff of the pediatric emergency department, the team involved forensic physicians (*N* = 4), dermatologists (*N* = 2), orthopedics (*N* = 2), radiologists (*N* = 3), gynaecologists (*N* = 2), oculists (*N* = 2), psychologists (*N* = 2) and psychiatrists (*N* = 2), who had also participated at the above-mentioned educational program. If a case of maltreatment was suspected, the forensic physician was available to come to the department within 60 min. The other specialists were available for consultation within 48 h according to the needs of each case. After the diagnostic workout, the diagnosis of maltreatment was confirmed or excluded by a consensus among the involved team members. The number of diagnosed cases in the period between January 2013 and December 2014 was compared with the number of diagnosed cases between January 2010 and December 2011 in the same pediatric emergency department. We excluded from the analysis cases diagnosed as neglect or sexual abuse because they were assessed accordingly with different management protocols. The study was performed in accordance with the principles stated in the Declaration of Helsinki and Good Clinical Practice.

Demographic data of the cases diagnosed as maltreatment in the two analysed periods are given in Table 1. From January 2013 to December 2014, a total of 53.788 patients were admitted to the pediatric emergency department of the Ca’ Granda, Ospedale Maggiore Policlinico, Milan (Italy). The mean age of the children was 2.1 ± 0.9 years (48.3 % males). In this period, 45 cases of maltreatment were diagnosed (33.3 % males, mean age 9.1 ± 3.6 years). During the period between January 2010 and December 2011, 54.599 children were admitted to pediatric emergency department (48.6 % males) and 39 (35.9 % males, mean age 8.5 ± 3.4 years) were diagnosed as maltreatment. An increased rate of diagnoses of 16.9 % was found during the period between January 2013 and December 2014.

**Table 1:** The table reports the demographic characteristics of cases diagnosed as maltreatment in the pediatric emergency department in the periods from January 2010 to December 2011 and from January 2013 to December 2014. Values are shown as frequency or as mean and range. No statistical difference was found between the two samples considered

Demographic characteristic of cases diagnosed as maltreatment		
	2010-2011	2013-2014
Cases, N	39	45
Male to Female ratio	0.56	0.5
Age, years	8.5 (0.2-16.5)	9.1 (0.2-17)
Cases born in Italy, N	14	21

In a retrospective survey involving ≈ 410 000 cases in 68 pediatric emergency units of 16 Italian regions, 6 per 10 000 were diagnosed as maltreatment [3]. In this survey, we found 7 per 10 000 cases diagnosed as maltreatment before the introduction of the educational program and the interdisciplinary team, and 8 per 10 000 after these interventions. Maltreatment cases are probably still under-diagnosed, even considering that the number of diagnosis has a high variability in the different Italian regions, with significant differences also among hospitals [4]. Increasing efforts should be taken to change this mindset, considering that families of maltreated children are often in critical situations and they could be, along with their child, the first beneficiaries of the child protective services involvement [5]. The study has some limitations. Firstly, the observational period is only 2 years long and therefore, the effect of the intervention is restricted to a short time-window. Secondly, data regarding the suspected (not confirmed) cases in the period before the introduction of the program were not available and, therefore, we could not compare such data. Finally, cases of sexual abuse and neglect were not analysed, because they were managed according to different protocols. In conclusion, preliminary results of the reported program are encouraging, but many efforts are still necessary to improve the child maltreatment identification. Implementation of educational programs, development of dedicated multidisciplinary teams and randomized trials to gauge their effectiveness can be important steps to face this challenge.
